# The Effect of Intra-Articular Injections of Hyaluronic Acid for the Treatment of Trapezio-Metacarpal Joint Osteoarthritis

**DOI:** 10.3390/jpm14080806

**Published:** 2024-07-30

**Authors:** Francesco Agostini, Elena Bressanin, Alessandro de Sire, Nikolaos Finamore, Federica Alviti, Valter Santilli, Andrea Bernetti, Marco Paoloni, Massimiliano Mangone

**Affiliations:** 1 Department of Anatomy, Histology, Forensic Medicine and Orthopedics, Sapienza University, 00185 Rome, Italy; francesco.agostini@uniroma1.it (F.A.); elena.bressanin@uniroma1.it (E.B.); federica.alviti@uniroma1.it (F.A.); marco.paoloni@uniroma1.it (M.P.); massimiliano.mangone@uniroma1.it (M.M.); 2 Department of Neurological and Rehabilitation Science, IRCCS San Raffaele, 00163 Rome, Italy; 3 Research Center on Musculoskeletal Health, MusculoSkeletalHealth@UMG, University of Catanzaro “Magna Graecia”, 88100 Catanzaro, Italy; alessandro.desire@unicz.it; 4 Physical and Rehabilitative Medicine Division, Department of Medical and Surgical Sciences, University of Catanzaro “Magna Graecia”, 88100 Catanzaro, Italy; 5 Department of Biological and Environmental Science and Technologies, University of Salento, 73100 Lecce, Italy; andrea.bernetti@unisalento.it

**Keywords:** hyaluronic acid, injections, osteoarthritis pain, rehabilitation, trapezio-metacarpal joint

## Abstract

Background: Osteoarthritis of the basal thumb joint is a debilitating disease with a high prevalence. Among pharmacological treatments, intra-articular injections of hyaluronic acid have been clinically applied. This study aimed to investigate the effectiveness and safety of intra-articular injections of hyaluronic acid for the treatment of trapezio-metacarpal joint osteoarthritis (TMOA), over a one-year period. Methods: Patients with TMOA were enrolled and treated with five consecutive intra-articular injections of hyaluronic acid (20 mg/2 mL, 500–750 KDa, HyalganBio) at weekly intervals. Primary outcomes were pain during different activities (changes in numerical rating scale) and function (pinch and grip strength), and secondary outcomes were safety (adverse events) and patient-reported outcomes (quick-DASH and SF-12). The outcomes were evaluated at baseline and 1-, 3-, 6-, and 12- months after the last injection. Results: A total of 29 patients were included. All participants completed the five injective sessions and the first follow-up. A total of 15 patients completed the study. During the follow-up period, intra-articular injections of hyaluronic acid have significantly reduced spontaneous and provoked pain and improved disability. No severe systemic adverse events were reported. Conclusions: At a follow-up of up to 12 months, patients with TMOA treated with intra-articular hyaluronic acid injections reported improvements in pain relief and quality of life.

## 1. Introduction

Osteoarthritis of the basal thumb joint, also known as carpometacarpal or trapezio-metacarpal (TMC) joint osteoarthritis (TMOA), or rhizarthrosis, is a highly debilitating disease of the middle and old-age population with a high global prevalence. Its clinical prevalence is double in women rather than in men (affecting 25% of post-menopausal women), while its radiographic prevalence is even higher, ranging from 45% to 60% [1,2].

Diagnosis of TMOA is based on the clinical examination. The main symptoms of TMOA are pain localized to the base of the thumb, tenderness, stiffness, loss of range of motion, and significant impairment of the entire hand function. The pain is often bilateral: it occurs in the ‘anatomical snuffbox’ and evolves in a fluctuating manner. The thumb is crucial for grasping and for the strength of the entire hand: functional impairment reduces the ability to perform activities of daily living, such as writing, opening a jar, turning a key, or handling small objects, ultimately reducing the patient’s quality of life [2,3,4]. 

Radiographic findings are commonly used to classify the stages of the disease, even if there is no clear correlation between clinical symptoms and the severity of the radiological changes [5].

Despite its high prevalence and disability, the therapeutic options for TMOA are still limited; its management usually requires a multidisciplinary approach using a combination of non-pharmacological, pharmacological, and surgical strategies [5,6]. Non-pharmacological measures include rest, activity modification, immobilization with splints or braces, ice packs, exercise, and physical therapies [7,8]. Pharmacological treatments include analgesics, topical or oral non-steroidal anti-inflammatory drugs (NSAIDs), and either corticosteroid (CS) or hyaluronic acid (HA) injections [1]. 

Surgical treatments, such as ligament reconstruction, tendon interposition, resection arthroplasty, and joint replacement or arthrodesis, are the last resort for patients with severe disability caused by OA who were failed by conservative treatments [9]. Among pharmacological treatments, intra-articular injections can be performed under ultrasound (US) guidance for a diagnostic or therapeutic purpose [10]. Several drugs can be injected into the TMC joint: HA injections are considered a safe therapeutic option for TMOA and have been clinically applied [2,9]. Injections of corticosteroids, platelet-rich plasma (PRP), prolotherapy, and stem cells are performed; treatments with infliximab, interferon-β, botulinum toxin, and oxygen–ozone have also been tried [11,12].

HA is a glycosaminoglycan (GAG) that is naturally found in synovial fluid. It is made up of a high number of disaccharides. Its effects on OA progression have been studied [13] and are related to the molecular weight, which may influence the signaling response [14,15].

However, the use of intra-articular (IA) injection therapy with HA is still a matter of debate, with differing views proposed by the American College of Rheumatology (ACR), which conditionally recommends OA glucocorticoid injections over other drug injections, and the European Alliance of Associations for Rheumatology (EULAR), which in its “future agenda” has identified the need to better determine the benefits of this treatment [16]. Furthermore, recent systematic reviews support the need for further evidence to establish the potential role of IA injections of HA on pain and function in patients with TMC OA [17].

In this study, we aimed to investigate the long-term effectiveness and safety of IA injections of HA for the treatment of TMOA patients.

## 2. Materials and Methods

### 2.1. Study Protocol and Study Design

This was a single-center, uncontrolled pilot study of the efficacy and safety of HA in patients with TMOA. The study protocol was submitted, reviewed, and approved by the local ethical committee of the “Sapienza” University of Rome, Italy (protocol number 247/2020, Approval no. 5885).

Outpatients in the Physical and Rehabilitation Department of the Umberto I University Hospital of Rome between March 2021 and May 2022 were screened and evaluated for inclusion. The study was conducted in accordance with the Declaration of Helsinki and in compliance with Good Clinical Practice guidelines.

Results have been reported following the “Consolidated Standards of Reporting Trials” guidelines (CONSORT 2010) [18].

### 2.2. Inclusion and Exclusion Criteria

Patients were considered eligible if they met all the following inclusion criteria:

○ they were aged 40 years old and above;
○ they had clinical symptoms for over 6 months (TMC joint pain, joint stiffness, decreased mobility, deformity, instability, functional impairment);
○ they had radiological observations indicative of TMOA (Eaton and Litter Stage II or III); and
○ they had a NRS score of pain ≥4 for over 6 months.

Patients who met one or more of the following exclusion criteria were not enrolled in the study:

○ they had a history of allergic reactions to an IA injection of HA;
○ they received IA injections of corticosteroids or other medical devices during the previous 3 months;
○ they underwent rehabilitation and physical therapies during the previous 4 weeks;
○ they were pregnant;
○ they had a heart pacemaker;
○ they had coagulopathy;
○ they had neurological degenerative and non-degenerative diseases;
○ they had a history of rheumatic or metabolic or other autoimmune diseases; or
○ they had a history of severe hand and thumb trauma.

### 2.3. Patients’ Screening, Baseline and Follow-Up Visits

Patients were evaluated for inclusion during a screening visit and then enrolled if they met the eligibility criteria. The screening visit was also considered the baseline visit for patients who were enrolled in the study. Individual demographic data, including age, sex, body weight, height, and affected hand, were collected. 

Patient medical and surgical histories, and radiological data were also evaluated at the baseline visit. Radiologic data were evaluated using the Eaton scale. The presence of local erythema, edema, and morning stiffness were also investigated. All participants were then administered HA injections once a week for 5 weeks within 15 days and were scheduled for follow-up visits at 1 month (T1), 3 months (T2), 6 months (T3), and and 12 months (T4) after the fifth injection. Assessment of primary and secondary outcomes was performed at baseline and at each follow-up point. A long-term follow-up period was chosen due to the lack of evidence of the long-term effectiveness of HA injections in TMOA, which has been suggested by other authors [19,20]. Participants were asked to fill in a diary to report their use of rescue analgesic medication. Rescue medication of 1 g of acetaminophen was allowed if needed in response to osteoarthritic pain. Data concerning concomitant medications and adverse events were collected at each visit. 

### 2.4. Therapeutic Intervention

All patients received five injections of HyalganBio (via a 20 mg/2 mL pre-filled syringe, molecular weight 500–730 KDa, manufactured by Fidia Farmaceutici S.p.A., Abano Terme, Italy), one week apart. All the injections were performed under ultrasound sterile guidance (10 MHz linear probe, Samsung HS60) and a vertical approach, as described by Orlandi et al. [21]. Following the drug administration, passive flexion and extension of the thumb were briefly performed to allow the diffusion of the injected fluid throughout the joint. Patients had to limit movements during the following 24 h and were instructed to wear a hand orthosis, using icepacks or acetaminophen 1 g to control the pain. The use of acetaminophen in this case did not count as rescue medication. 

### 2.5. Outcomes and Follow-Up Periods

The primary outcomes were the assessment of pain and function, and the secondary outcomes included the assessment of safety, disability, and quality of life. Assessments of both primary and secondary outcomes were performed at baseline (T0), and at 1 month (T1), 3- (T2), 6- (T3), and 12 months (T4) after the fifth injection.

#### 2.5.1. Primary Outcomes—Pain and Function

Pain was assessed according to changes in the numerical pain rating scale (NRS), which assesses a patient’s pain from 0 to 10, with 0 being “no pain” and 10 indicating the greatest pain intensity. In our study, we investigated changes in the NRS scale during different activities: pain at rest, pain evoked during movement, night pain, and mean pain suffered during the day.

Function was assessed by evaluating the pinch strength tests (lateral pinch, two-point pinch test, and three-point pinch test) and the handgrip strength test. Each test was performed to evaluate both the affected and the unaffected side, to compare treated versus untreated hand function. The pinch and grip strength of the hand were assessed at the Jamar and Pinch Gauges hydraulic dynamometers. 

#### 2.5.2. Secondary Outcomes—Adverse Events and Disability/Quality of Life

Safety and tolerability of the treatment were assessed by recording adverse events, both immediately after each injection and at follow-up visits.

Disability and quality of life were assessed using two questionnaires: Quick-DASH and the 12-Item Short Form Health Survey (SF-12). Quick-DASH is an abbreviated version of the original DASH (Disabilities of the Arm, Shoulder, and Hand) outcome measure. It is an 11-item questionnaire that measures an individual’s ability to complete tasks and the severity of symptoms [22]. The SF-12 is a self-reported outcome measure assessing the impact of health on an individual’s everyday life. The SF-12 is a shortened version of its predecessor, the SF-36, which performs a generic and multipurpose health assessment. The subject is asked to answer questions about how he is able to perform his usual activities (up to 4 weeks before the day of compilation of the questionnaire), and these answers allow to calculate two summary scores of physical and mental health status: the Physical Component Summary (PCS) and a Mental Component Summary (MCS) [23,24,25].

### 2.6. Calculation of Sample Size

The sample size was calculated using the G*Power version 3.1.9.7, considering the quick-DASH scale variation, through an analysis that compared the mean values for paired data. According to Gummesson C. et al. [22], we used the following values: 35 ± 22 and 24 ± 23. We considered a statistical power 1-beta of 0.80 and an α significance of 0.05. With these parameters, the required sample size was equal to 28 subjects.

### 2.7. Statistical Analysis

Statistical analysis with an intention-to-treat approach was performed using the IBM SPSS Statistics version 29 software. Descriptive statistics were used to describe the collected data: continuous data were expressed in mean/median ± standard deviation (SD)/interquartile range (IR), while categorical data were expressed in number and frequency. The normal distribution was tested with the Shapiro–Wilk test. The Friedman test was performed to compare the values of the variations over time of the considered parameters. Post-hoc analysis with Bonferroni correction was carried out to determine the factors of significance. A *p* value of <0.05 was considered statistically significant. 

## 3. Results

**Figure 1:**
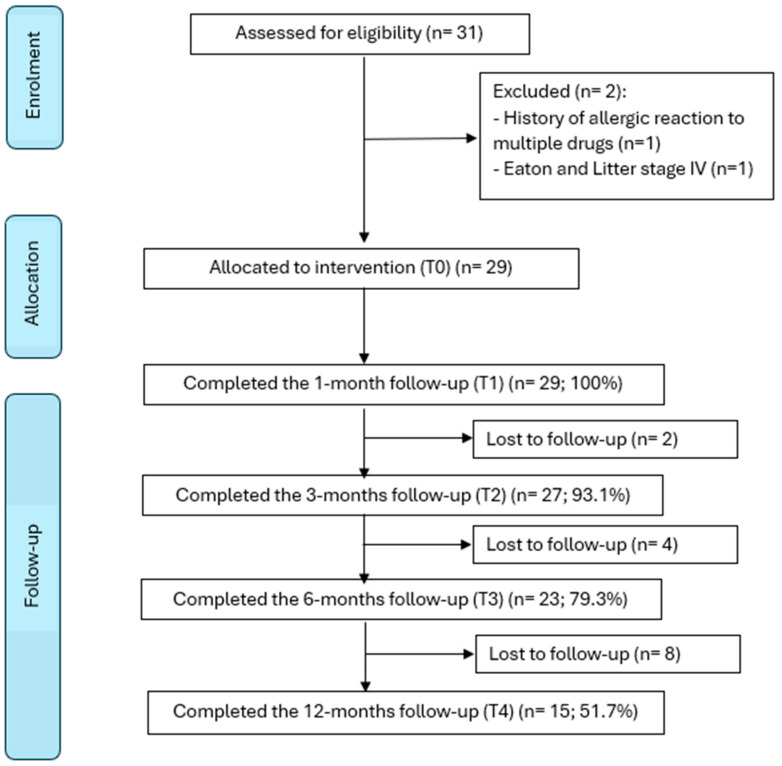
Study flowchart.

After screening a total of 31 participants, 29 patients who met the inclusion criteria were finally included in the study. The flow diagram in Figure 1 summarizes the selection process, including the reasons for exclusion.

The recruitment phase started in 2021, with the first participant recruited on March 30th, and ended in 2022, with the last participant recruited on May 27th. All participants were recruited during a screening visit (baseline, T0); during this visit, the investigators evaluated patient eligibility for inclusion. Patients underwent the cycle of five injections and subsequent follow-ups.

The characteristics of the study patients at baseline are shown in Table 1. 

All the participants (100%) completed the five injective sessions and the first follow-up (at 1 month after the fifth injection). At T2 (the 3-month follow-up), two participants (6.89%) were lost to follow-up; at T3 (the 6-month follow-up), six participants (20.68%) were lost to follow-up; at T4 (the 1-year follow-up), fourteen participants (48.27%) were lost to follow-up. All patients who dropped out of the study did so for personal reasons or because of low compliance with the study.

Table 2, Table 3 and Table 4 summarize the evaluations of the outcomes of the study performed at baseline (T0) and at each follow-up. 

### 3.1. Primary Outcomes—Pain and Function

**Figure 2:**
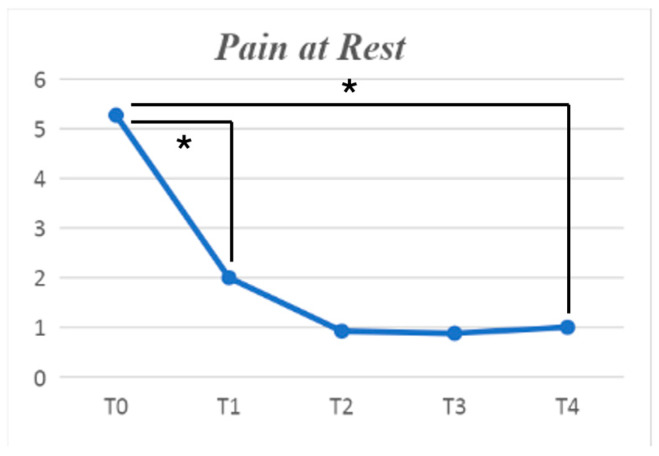
Mean pain at rest, measured using the numerical pain rating scale and its improvement in time. The symbol * indicates a statistically significant difference between the follow-ups linked by the row.

**Figure 3:**
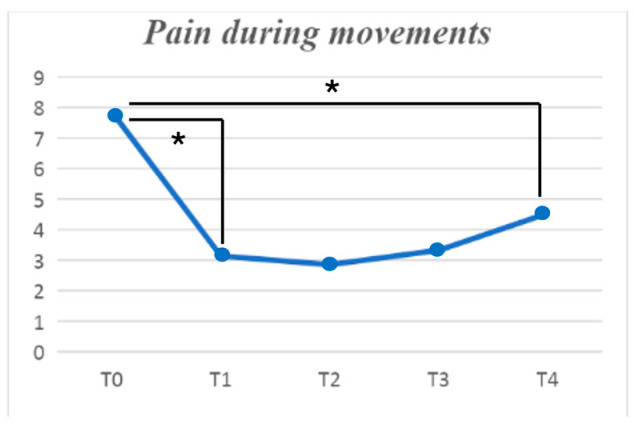
Mean pain during movement, measured using the numerical pain rating scale and its improvement in time. The symbol * indicates a statistically significant difference between the follow-ups linked by the row.

A statistically significant effectiveness of the treatment on all the pain subsets was found. When considering mean pain during the day, pain at rest, and pain at movement, a statistically significant effectiveness was found (*p* < 0.001), with the strongest results obtained between baseline and one-month follow-up. There was also a reduction of the pain at night (*p* = 0.006), which was also more robust during the first month after the treatment (Figure 2 and Figure 3).

All of these results were obtained when comparing T1 to baseline; no further statistically significant improvements were found when comparing the other follow-ups to T1.

No statistically significant improvements were found in the functional outcomes.

### 3.2. Secondary Outcomes—Safety and Disability/Quality of Life Perception

A statistically significant reduction in quick-DASH score (*p* < 0.001) was found, with the major results obtained at one-month follow-up. 

**Figure 4:**
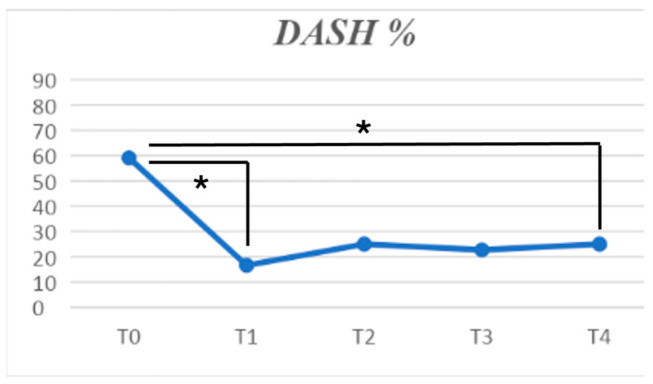
DASH reduction at different follow-ups. The symbol * indicates a statistically significant difference between the follow-ups linked by the row.

**Figure 5:**
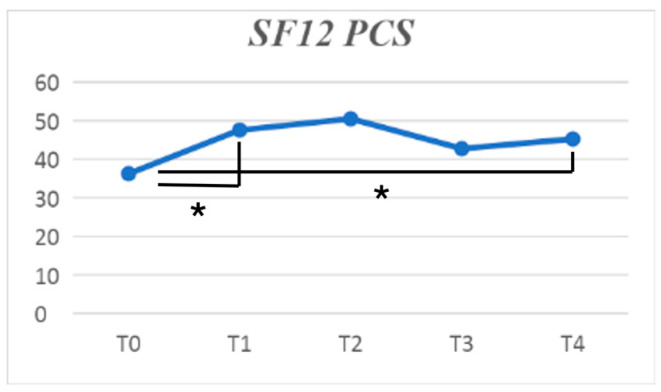
SF-12 (physical component summary) improvement at different follow-ups. The symbol * indicates a statistically significant difference between the follow-ups linked by the row.

Furthermore, the physical component summary subscale of SF-12 improved (*p* = 0.034), with the major effectiveness between baseline and T1. No differences could be found in the mental component score subscale of SF-12 (Figure 4 and Figure 5).

No severe adverse vents (AEs) were reported. The most frequent adverse event was transient pain at the site of injection.

## 4. Discussion

TMOA is a chronic condition with remissions and exacerbations [19]. Osteoarthritis involves the joint, with degradation of the articular cartilage, subchondral sclerosis, and hyperplasia of the synovial tissue [26]. Destructive processes of articular cartilage play a pivotal role in the development and progression of this disease, resulting from an imbalance between catabolic and anabolic events [27].

Non-surgical treatments should be the first line of therapy, as they can play a role in treating symptoms but do not influence function or strength [28]. A recent paper by the American Society of Hand Therapists [29] described several non-surgical therapeutic modalities, which might have positive outcomes for long periods (more than 5 years) but are often underestimated and under-prescribed by non-specialists [20]. Specifically, orthoses are recommended during painful functional tasks [19,29]. Topical non-steroidal anti-inflammatory drugs (NSAIDs) also proved effective for treating pain [30]. Systemic steroids and NSAIDs can provide temporary pain relief and inhibit further inflammation but are associated with significant side effects when administered over a long period of time [4]. However, none of the currently used treatments has a significant impact on preventing or retarding disease progression [28], and no drugs are currently available with disease-modifying properties [31,32].

Proteoglycans, also conjugated with glycosaminoglycans, are fundamental components of the extracellular matrix of the articular cartilage, and osteoarthritic joints show a reduction in proteoglycan levels [33,34]. Among glycosaminoglycans, HA is a high-molecular-weight polysaccharide widely distributed in the connective tissue extracellular matrix: it is normally formed in the synovial fluid of joints and plays an important role in the biomechanics of the synovial fluid by enhancing the viscoelastic and lubricating functions of the joints [35,36]. Intra-articular drug administration is a way of administration for patients with joint inflammation and osteoarthritis, and HA is one of the medicines that can be effectively administered via this method [35,36,37,38,39,40]. Viscosupplementation with HA may be prescribed to replenish the concentration and to restore the rheological properties of the synovial fluid [34]. Experimental models also reported the effects of proteoglycan administration in patients with OA [33].

The effectiveness of intra-articular injections of HA in hand osteoarthritis is still a subject of debate caused by the great heterogeneity of the experiments carried out in this field [9,41]. When compared to placebo, its superiority to placebo has been variable [30,42]. One of the problems is the inconsistency of how HA is administered, with several studies also comparing different protocols of administration and different HA formulations to assess its effectiveness [43].

To date, there are no standardized treatment protocols for thumb OA. In the studies carried out, protocols mainly differ in the molecular weight of the HA used, the number of injections given (from one to five), the methods of administering the injections (blind or ultrasound-guided), and the variability in the stage of the arthritic pathology [2,44]. 

The present study administered five IA injections of HA (20 mg/2 mL, 500–750 KDa, HyalganBio). These injections were conducted under ultrasound guidance in accordance with the indications of the EUROVISCO 2020 group. This group strongly recommends, for the TMC joint, the use of a guide for diagnostic imaging and during intra-articular injections. This is because ultrasound guidance is useful not only to verify the optimal positioning of the needle but also to inject the optimal volume into very small joints [45,46]. This has previously been reported by Di Sante et al., 2011 [47]. Although the present study reports benefits with the administration of five IA injections, it should be considered that TMC injections can cause discomfort during the procedure, and the high number of administrations is not optimal. 

In contrast to the systematic review by Kroon et al. [30], which reported no improvements in pain or function in patients treated with HA, we found improvements in all the pain subscales evaluated. Most of the pain reduction is achieved during the first follow-up (up to 1 month after the fifth injection), but at the 6-month follow-up it is still statistically superior to baseline. Data at one-year follow-up were promising but should be carefully evaluated because of the high number of patients lost to follow-up. 

Our findings are in accordance with those of Frizziero et al., who also reported a subsequent initial decrease in the effect of the treatment at the 3-month follow-up [48]. It was also interesting to note that 14 of the 29 patients were lost to follow-up. Unfortunately, it was not possible to contact these patients to understand possible motivations and to obtain information about possible surgery or other treatments. 

Regarding secondary outcomes, there is a lack of consensus on which outcomes should be used to evaluate the disability related to TMOA [49].

The quick-DASH and SF-12 scales were chosen to evaluate the disability and the quality of life, and a statistically significant improvement was found in both (except for the mental component score of the SF-12). Quick-DASH is a region-specific patient-reported outcome measure that can be influenced by shoulder or elbow conditions [50]. Comorbidities (including depressive symptoms) should also be taken into consideration when evaluating the SF-12 MCS score because they are associated with a worse evaluation by the patient of their own hand function [50].

Furthermore, it was interesting to note that no patients needed to take any analgesic therapy during the observation period, eliminating possible side effects linked to systemic use of drugs.

Finally, our study did not report any severe/systemic adverse effects. This is in accordance with the data reported in the literature [2,9].

### Limitations of the Present Study

The first limit is the absence of a control group; therefore, hyaluronic acid injections were not compared with a standard-of-care treatment. Another limitation is that several physicians took part in the evaluations. Although we mainly chose patient-reported outcome measures, a different explication of the scales administered by the operators might have a small effect on the reported results. Moreover, the small number of patients, with the high number of lost to follow-up at 1 year, hampered every possible conclusion drawn at the 1-year follow-up.

## 5. Conclusions

Patients with TMOA in this study treated with HA injections reported a reduction in pain. With the limitations of a high dropout rate and the lack of a control group, the improvement appears to be prolonged and sustained over a twelve-month observation period, in line with the reduction in patient-perceived disability reported in our results. No severe complications were reported. This might be considered for patients who are unwilling to undergo surgery or for whom surgery is contraindicated. The results of this study suggest that the IA treatment with HA could be considered a therapeutic choice in the treatment of patients with osteoarthritis of the TMC joint in the short-, medium-, and long-term. Further studies with larger samples and controls may help to confirm the results.

## Figures and Tables

**Table 1:** Demographic characteristics of the participants at baseline.

Characteristics of the Participants at Baseline	Participants (*n* = 29)
Sex, M/F (%M)	6/23 (20.68%)
Age (mean ± SD)	65.0 ± 8.7 years
Body weight (mean ± SD)	65.5 ± 12.0 kg
Body height (mean ± SD)	157.0 ± 9.2 cm
Side of the affected hand, left/right (%Left)	16/13 (55.17%)
Dominant hand, left/right (%Left)	1/28 (3.45%)

SD = standard deviation.

**Table 2:** Pain at baseline and during the follow-up visits.

	T0	T1	T2	T3	T4	ANOVA *p*-Value
	Participants (*n* = 29)	Participants (*n* = 29)	Participants (*n* = 27)	Participants (*n* = 23)	Participants (*n* = 15)	
Pain at rest, NRS (mean ± SD)	5.31 ± 1.87	1.07 ± 1.71	0.93 ± 1.84	1.09 ± 2.04	1.00 ± 2.24	<0.001
Pain during movements, NRS (mean ± SD)	7.86 ± 1.66	3.14 ± 2.86	2.85 ± 2.78	3.32 ± 3.15	4.53 ± 3.09	<0.001
Night pain, NRS (mean ± SD)	2.07 ± 3.12	0.45 ± 1.35	0.41 ± 1.53	0.83 ± 1.99	0.93 ± 2.49	0.006
Mean pain during the day, NRS (mean ± SD)	5.48 ± 1.81	1.17 ± 1.75	1.22 ± 2.15	1.61 ± 2.59	1.53 ± 2.47	<0.001
Tenderness before injections, Yes/No (%Yes)	2/29 (6.9%)	0/29 (0%)	0/27 (0%)	0/23 (0%)	0/15 (0%)	

SD = standard deviation; NRS = numerical rating scale.

**Table 3:** Pinch/handgrip strength tests at baseline and during the follow-up visits.

	T0	T1	T2	T3	T4	ANOVA *p*-Value
Lateral pinch (kg), mean (±SD)	6.15 ± 2.23	6.78 ± 2.24	6.47 ± 1.98	6.60 ± 2.21	6.40 ± 2.48	0.693
Three-point pinch (kg), mean (±SD)	5.28 ± 1.73	6.06 ± 1.98	5.96 ± 1.87	5.52 ± 2.12	5.85 ± 2.05	0.061
Two-point pinch (kg), mean (±SD)	4.09 ± 1.36	4.69 ± 1.30	4.66 ± 1.45	4.00 ± 1.58	4.80 ± 1.31	0.060
Handgrip (kg), mean (±SD)	25.76 ± 9.91	28.86 ± 9.66	28.37 ± 9.07	28.27 ± 9.55	26.55 ± 9.21	0.071

SD = standard deviation.

**Table 4:** Patient-reported outcome measures of the participants at baseline and during the follow-up visits.

	T0	T1	T2	T3	T4	ANOVA *p*-Value
Quick-DASH (mean ± SD)	50.86 ± 19.91	22.02 ± 16.29	22.9 ± 19.25	13.18 ± 20.03	35.62 ± 23.97	<0.001
SF-12 PCS (mean ± SD)	39.73 ± 7.10	45.33 ± 7.97	47.52 ± 8.03	44.26 ± 9.23	44.10 ± 7.91	0.034
SF-12 MCS (mean ± SD)	46.06 ± 11.00	50.41 ± 11.92	52.05 ± 9.81	51.39 ± 8.50	49.83 ± 7.14	0.15

SD = standard deviation, SF-12 = 12-item short form health survey, PCS = physical component summary, MCS = mental component summary.

## Data Availability

Data are contained within the article.

## References

[1] Cormier G., Le Goff B., Denis A., Varin S., Auzanneau L., Dimet J., Le Thuaut A. (2019). Corticosteroids injections versus corticosteroids with hyaluronic acid injections in rhizarthrosis: The randomised multicentre RHIZ’ART trial study protocol. BMJ Open..

[2] Tenti S., Cheleschi S., Mondanelli N., Giannotti S., Fioravanti A. (2021). New Trends in Injection-Based Therapy for Thumb-Base Osteoarthritis: Where Are We and where Are We Going?. Front. Pharmacol..

[3] Spielman A.F., Sankaranarayanan S., Lessard A.S. (2022). Joint Preserving Treatments for Thumb CMC Arthritis. Hand Clin..

[4] Hasiba-Pappas S., Kamolz L.P., Luze H., Nischwitz S.P., Lumenta D.B., Winter R. (2023). Regenerative Therapies for Basal Thumb Arthritis-A Systematic Review. Int. J. Mol. Sci..

[5] Wilkens S.C., Meghpara M.M., Ring D., Coert J.H., Jupiter J.B., Chen N.C. (2019). Trapeziometacarpal Arthrosis. JBJS Rev..

[6] Bakri K., Moran S.L. (2015). Thumb carpometacarpal arthritis. Plast. Reconstr. Surg..

[7] Pickrell B.B., Eberlin K.R. (2019). Thumb Basal Joint Arthritis. Clin. Plast. Surg..

[8] Villafañe J.H., Valdes K., Pedersini P., Berjano P. (2019). Thumb carpometacarpal osteoarthritis: A musculoskeletal physiotherapy perspective. J. Bodyw. Mov. Ther..

[9] Wang P.H., Wu C.H., Ma C.H., Chiu Y.C., Wu P.T., Jou I.M. (2023). Comparison of intra-articular injection of ArtiAid^®^-Mini with Ostenil^®^-Mini for trapeziometacarpal osteoarthritis: A double-blind, prospective, randomized, non-inferiority trial. Jt. Dis. Relat. Surg..

[10] Tortora S., Messina C., Albano D., Serpi F., Corazza A., Carrafiello G., Sconfienza L.M., Gitto S. (2021). Ultrasound-guided musculoskeletal interventional procedures around the elbow, hand and wrist excluding carpal tunnel procedures. J. Ultrason..

[11] Oo W.M., Hunter D.J. (2023). Efficacy, Safety, and Accuracy of Intra-articular Therapies for Hand Osteoarthritis: Current Evidence. Drugs Aging.

[12] de Sire A., Marotta N., Sconza C., Lippi L., Ferrante V.D., Respizzi S., Invernizzi M., Ammendolia A. (2024). Oxygen-ozone therapy for pain relief in patients with trapeziometacarpal osteoarthritis: A proof-of-concept study. Disabil. Rehabil..

[13] Valachová K., Šoltés L. (2021). Hyaluronan as a Prominent Biomolecule with Numerous Applications in Medicine. Int. J. Mol. Sci..

[14] Turley E.A., Noble P.W., Bourguignon L.Y. (2002). Signaling properties of hyaluronan receptors. J. Biol. Chem..

[15] Balazs E.A. (2003). Analgesic effect of elastoviscous hyaluronan solutions and the treatment of arthritic pain. Cells Tissues Organs.

[16] Pinto I., Duarte C., Vilabril F., Brito I. (2022). Impact of Hyaluronic Acid Treatment on Rhizarthrosis: A Systematic Review. ARP Rheumatol..

[17] Tran K., Loshak H. (2019). Intra-Articular Hyaluronic Acid for Viscosupplementation in Osteoarthritis of the Hand, Shoulder, and Temporomandibular Joint: A Review of Clinical Effectiveness and Safety [Internet].

[18] Schulz K.F., Altman D.G., Moher D., CONSORT Group (2010). CONSORT 2010 Statement: Updated guidelines for reporting parallel group randomised trials. J. Clin. Epidemiol..

[19] Spaans A.J., van Minnen L.P., Kon M., Schuurman A.H., Schreuders A.R., Vermeulen G.M. (2015). Conservative treatment of thumb base osteoarthritis: A systematic review. J. Hand Surg. Am..

[20] Esteban Lopez L.M.J., Hoogendam L., Vermeulen G.M., Tsehaie J., Slijper H.P., Selles R.W., Wouters R.M., The Hand-Wrist Study Group (2023). Long-Term Outcomes of Nonsurgical Treatment of Thumb Carpometacarpal Osteoarthritis: A Cohort Study. J. Bone Jt. Surg. Am..

[21] Orlandi D., Corazza A., Silvestri E., Serafini G., Savarino E.V., Garlaschi G., Mauri G., Cimmino M.A., Sconfienza L.M. (2014). Ultrasound-guided procedures around the wrist and hand: How to do. Eur. J. Radiol..

[22] Gummesson C., Ward M.M., Atroshi I. (2006). The shortened disabilities of the arm, shoulder and hand questionnaire (QuickDASH): Validity and reliability based on responses within the full-length DASH. BMC Musculoskelet. Disord..

[23] Kodraliu G., Mosconi P., Groth N., Carmosino G., Perilli A., Gianicolo E.A., Rossi C., Apolone G. (2001). Subjective health status assessment: Evaluation of the Italian version of the SF-12 Health Survey. Results from the MiOS Project. J. Epidemiol. Biostat..

[24] Ware J.E. (2000). SF-36 health survey update. Spine.

[25] Ware J., Kosinski M., Keller S.D. (1996). A 12-Item Short-Form Health Survey: Construction of scales and preliminary tests of reliability and validity. Med. Care.

[26] Coaccioli S., Sarzi-Puttini P., Zis P., Rinonapoli G., Varrassi G. (2022). Osteoarthritis: New Insight on Its Pathophysiology. J. Clin. Med..

[27] Goldring S.R., Goldring M.B. (2016). Changes in the osteochondral unit during osteoarthritis: Structure, function and cartilage-bone crosstalk. Nat. Rev. Rheumatol..

[28] Cai X., Yuan S., Zeng Y., Wang C., Yu N., Ding C. (2021). New Trends in Pharmacological Treatments for Osteoarthritis. Front. Pharmacol..

[29] Algar L., Naughton N., Ivy C., Loomis K., McGee C., Strouse S., Fedorczyk J. (2023). Assessment and treatment of nonsurgical thumb carpometacarpal joint osteoarthritis: A modified Delphi-based consensus paper of the American Society of Hand Therapists. J. Hand Ther..

[30] Kroon F.P.B., Carmona L., Schoones J.W., Kloppenburg M. (2018). Efficacy and safety of non-pharmacological, pharmacological and surgical treatment for hand osteoarthritis: A systematic literature review informing the 2018 update of the EULAR recommendations for the management of hand osteoarthritis. RMD Open.

[31] Kloppenburg M., Kroon F.P., Blanco F.J., Doherty M., Dziedzic K.S., Greibrokk E., Haugen I.K., Herrero-Beaumont G., Jonsson H., Kjeken I. (2019). 2018 update of the EULAR recommendations for the management of hand osteoarthritis. Ann. Rheum. Dis..

[32] Bernetti A., Agostini F., Paoloni M., Raele M.V., Farì G., Megna M., Mangone M. (2023). Could Hyaluronic Acid Be Considered as a Senomorphic Agent in Knee Osteoarthritis? A Systematic Review. Biomedicines.

[33] Ajeeshkumar K.K., Vishnu K.V., Navaneethan R., Raj K., Remyakumari K.R., Swaminathan T.R., Suseela M., Asha K.K., Sreekanth G.P. (2019). Proteoglycans isolated from the bramble shark cartilage show potential anti-osteoarthritic properties. Inflammopharmacology.

[34] More S., Kotiya A., Kotia A., Ghosh S.K., Spyrou L.A., Sarris I.E. (2020). Rheological properties of synovial fluid due to viscosupplements: A review for osteoarthritis remedy. Comput. Methods Programs Biomed..

[35] Bernetti A., Mangone M., Paolucci T., Santilli V., Verna S., Agostini F., Paoloni M. (2020). Evaluation of the efficacy of intra-articular injective treatment with reticular hyaluronic acid (Mo.Re. Technology) in amateur athletes with over-use gonarthrosis. Med. Sport.

[36] Castro-Calderón A., Roccuzzo A., Ferrillo M., Gada S., González-Serrano J., Fonseca M., Molinero-Mourelle P. (2022). Hyaluronic acid injection to restore the lost interproximal papilla: A systematic review. Acta Odontol. Scand..

[37] Agostini F., Ferrillo M., Bernetti A., Finamore N., Mangone M., Giudice A., Paoloni M., de Sire A. (2023). Hyaluronic acid injections for pain relief and functional improvement in patients with temporomandibular disorders: An umbrella review of systematic reviews. J. Oral Rehabil..

[38] Lippi L., Ferrillo M., Turco A., Folli A., Moalli S., Refati F., Perrero L., Ammendolia A., de Sire A., Invernizzi M. (2023). Multidisciplinary Rehabilitation after Hyaluronic Acid Injections for Elderly with Knee, Hip, Shoulder, and Temporomandibular Joint Osteoarthritis. Medicina.

[39] Familiari F., Ammendolia A., Rupp M.C., Russo R., Pujia A., Montalcini T., Marotta N., Mercurio M., Galasso O., Millett P.J. (2023). Efficacy of intra-articular injections of hyaluronic acid in patients with glenohumeral joint osteoarthritis: A systematic review and meta-analysis. J. Orthop. Res..

[40] Snetkov P., Zakharova K., Morozkina S., Olekhnovich R., Uspenskaya M. (2020). Hyaluronic Acid: The Influence of Molecular Weight on Structural, Physical, Physico-Chemical, and Degradable Properties of Biopolymer. Polymers.

[41] Bernetti A., Agostini F., Alviti F., Giordan N., Martella F., Santilli V., Paoloni M., Mangone M. (2021). New Viscoelastic Hydrogel Hymovis MO.RE. Single Intra-articular Injection for the Treatment of Knee Osteoarthritis in Sportsmen: Safety and Efficacy Study Results. Front. Pharmacol..

[42] Veronese N., Smith L., Bolzetta F., Cester A., Demurtas J., Punzi L. (2021). Efficacy of conservative treatments for hand osteoarthritis: An umbrella review of interventional studies. Wien. Klin. Wochenschr..

[43] Perugia D., Lanzetti R., Vetrano M., Vavala C., Pascali S., Nusca S.M., Santoboni F., Vulpiani M.C. (2020). Mind Term Effects of a Single Injecton versus three Injecton of Hyaluronic Acid in Patients with Rhizarthrosis. Muscle Ligaments Tendons J..

[44] Monfort J., Rotés-Sala D., Segalés N., Montañes F.J., Orellana C., Llorente-Onaindia J., Mojal S., Padró I., Benito P. (2015). Comparative efficacy of intra-articular hyaluronic acid and corticoid injections in osteoarthritis of the first carpometacarpal joint: Results of a 6-month single-masked randomized study. Jt. Bone Spine.

[45] Umphrey G.L., Brault J.S., Hurdle M.F., Smith J. (2008). Ultrasound-guided intra-articular injection of the trapeziometacarpal joint: Description of technique. Arch. Phys. Med. Rehabil..

[46] Conrozier T., Raman R., Chevalier X., Henrotin Y., Monfort J., Diraçoglù D., Bard H., Baron D., Jerosch J., Richette P. (2021). Viscosupplementation for the treatment of osteoarthritis. The contribution of EUROVISCO group. Ther. Adv. Musculoskelet. Dis..

[47] Di Sante L., Cacchio A., Scettri P., Paoloni M., Ioppolo F., Santilli V. (2011). Ultrasound-guided procedure for the treatment of trapeziometacarpal osteoarthritis. Clin. Rheumatol..

[48] Frizziero A., Maffulli N., Masiero S., Frizziero L. (2014). Six-months pain relief and functional recovery after intra-articular injections with hyaluronic acid (mw 500–730 KDa) in trapeziometacarpal osteoarthritis. Muscles Ligaments Tendons J..

[49] Copeland A., Gallo L., Weber C., Moltaji S., Gallo M., Murphy J., Axelrod D., Thoma A. (2021). Reporting Outcomes and Outcome Measures in Thumb Carpometacarpal Joint Osteoarthritis: A Systematic Review. J. Hand Surg. Am..

[50] Calfee R., Chu J., Sorensen A., Martens E., Elfar J. (2015). What Is the Impact of Comorbidities on Self-rated Hand Function in Patients With Symptomatic Trapeziometacarpal Arthritis?. Clin. Orthop. Relat. Res..

